# Prevalence of undiagnosed metabolic syndrome using three different definitions and identifying associated risk factors among apparently healthy adults in Karachi, Pakistan: a cross-sectional survey in the year 2022

**DOI:** 10.1186/s13690-024-01250-3

**Published:** 2024-02-20

**Authors:** Syed Omair Adil, Kamarul Imran Musa, Fareed Uddin, Asima Khan, Irfanullah Khan, Areebah Shakeel, Kashif Shafique, Md Asiful Islam

**Affiliations:** 1 Department of Community Medicine, School of Medical Sciences, Universiti Sains Malaysia (USM), Health Campus, 16150 Kubang Kerian, Kelantan Malaysia; 2 School of Public Health, Dow University of Health Sciences (DUHS), 74200 Karachi, Pakistan; 3 Department of Community Medicine, Dow International Medical College, DUHS, 74200 Karachi, Pakistan; 4 Public Health Department, Baqai Institute of Diabetology & Endocrinology, 75340 Karachi, Pakistan; 5 Discipline of Clinical Pharmacy, School of Pharmaceutical Sciences, USM, 11800 Penang, Malaysia; 6 Chronic Kidney Disease Resource Centre, School of Medical Sciences, Health Campus, USM, 16150 Kubang Kerian, Kelantan Malaysia; 7 Department of Research, Children Hospital, 75300 Karachi, Pakistan; 8 WHO Collaborating Centre for Global Women’s Health, Institute of Metabolism and Systems Research, University of Birmingham, B15 2TT Birmingham, UK

**Keywords:** Prevalence, Metabolic syndrome, Healthy individuals, Karachi, Pakistan

## Abstract

**Objective:**

To determine the prevalence and associated risk factors of undiagnosed metabolic syndrome (MetS) using three different definitions among apparently healthy adults of Karachi, Pakistan.

**Methods:**

This community-based cross-sectional survey was conducted in Karachi, Pakistan, from January 2022 to August 2022. A total of 1065 healthy individuals aged 25–80 years of any gender were consecutively included. MetS was assessed using the National Cholesterol Education Program for Adult Treatment Panel (NCEP-ATP) III guidelines, International Diabetes Federation (IDF), and modified NCEP-ATP III.

**Results:**

The prevalence of MetS was highest with the modified NCEP-ATP III definition at 33.9% (95% CI: 31–36), followed by the IDF definition at 32.2% (95% CI: 29–35). In contrast, the prevalence was lower at 22.4% (95% CI: 19–25) when using the NCEP ATP III definition. The risk of MetS significantly increases with higher BMI, as defined by the IDF criteria (adjusted OR [ORadj] 1.13, 95% CI 1.09–2.43), NCEP-ATP III criteria (ORadj 1.15, 95% CI 1.11–1.19), and modified NCEP-ATP III criteria (ORadj 1.16, 95% CI 1.12–1.20). Current smokers had significantly higher odds of MetS according to the IDF (ORadj 2.72, 95% CI 1.84–4.03), NCEP-ATP III (ORadj 3.93, 95% CI 2.55–6.06), and modified NCEP-ATP III (ORadj 0.62, 95% CI 0.43–0.88). Areca nut use was associated with higher odds of MetS according to both IDF (ORadj 1.71, 95% CI 1.19–2.47) and modified NCEP-ATP III criteria (ORadj 1.58, 95% CI 1.10–2.72). Furthermore, low physical activity had significantly higher odds of MetS according to the NCEP-ATP III (ORadj 1.36, 95% CI 1.01–1.84) and modified NCEP-ATP III criteria (ORadj 1.56, 95% CI 1.08–2.26).

**Conclusion:**

One-third of the healthy individuals were diagnosed with MetS based on IDF, NCEP-ATP III, and modified NCEP-ATP III criteria. A higher BMI, current smoking, areca nut use, and low physical activity were significant factors.

**Supplementary Information:**

The online version contains supplementary material available at 10.1186/s13690-024-01250-3.

**Table:** 

**Text box 1. Contributions to the literature**
• By using three internationally recognized criteria, this community-based study has found that a higher number of adult individuals who were perceiving themselves as healthy had diseases such as diabetes, hypertension, and abnormal lipid profile.
• The study findings showed that being overweight/obese, use of substance abuse such as smoking and areca nut, and low physical activity increase the risk of these diseases.
• The study has highlighted the importance of public health screening of apparently healthy individuals for metabolic syndrome. The prompt screening and intervention may also prevent the future risk of cardiovascular disease in these individuals.

## Introduction

Metabolic syndrome (MetS) is a cluster of metabolic disorders that includes abdominal obesity, high blood pressure, high fasting blood sugar, high triglycerides (TG), and low high-density lipoprotein (HDL) cholesterol [1]. These conditions often occur together and increase the risk of developing cardiovascular disease, stroke, and type 2 diabetes [1, 2]. Moreover, factors that trigger the risk of non-communicable diseases, such as physical inactivity, disturbance of sleep patterns, intake of an unhealthy diet, and stress, have increased the already burdened prevalence of diseases worldwide [3–5].

MetS has multiple definitions, including those from the World Health Organization (WHO) [6], Adult Treatment Panel III of the National Cholesterol Education Program (NCEP-ATP III) [7], International Diabetes Federation (IDF) [8], and a consensus between the American Heart Association and the National Heart, Lung, and Blood Institute (AHA/NHLBI) and IDF (Join Interim Statement) [9]. The WHO and IDF definitions are primarily centered around glucose metabolism and obesity, respectively, while the NCEP-ATP III definition mainly focused on predicting cardiovascular disease [10]. For most of these criteria, MetS is diagnosed based on the presence of at least three of the five abovementioned components, with some variations in diagnostic cut-offs [11].

The prevalence of MetS varies across different populations and depends on the criteria used in other definitions [10]. A recently published meta-analysis on the global general population of adults has reported that MetS global prevalence varied from 12.5 to 31.4%, depending on the diagnostic criteria.

Like many other countries, the burden of MetS is increasing remarkably in Pakistan [12, 13]. As a middle-income country with a weak healthcare infrastructure and a population of around 208 million, Pakistan is facing a significant impact on its already overburdened healthcare resources [14]. Earlier studies reported that healthy Pakistani adults who are above 40 years of age are more vulnerable and have more chances of developing risk factors associated with MetS and cardiovascular disease [15, 16]. Thus, a study that screens the healthy at-risk adult population is the need of the hour. Furthermore, a recent in-depth literature search revealed that reporting of MetS in most of the previously published Pakistani studies is based on a single criterion, i.e., either NCEP-ATP III or IDF guidelines [12, 15–18]. There is a dire need for a study that estimates the prevalence of undiagnosed MetS in persons who appear to be in good health using various international recommendations. Identifying the risk factors for MetS will help policy makers in Pakistan with their guideline for disease control. Therefore, this study was planned to evaluate the prevalence of undiagnosed MetS using three widely used screening criteria among apparently healthy adults residing in Karachi, Pakistan. Furthermore, the study also reported the determinants that can contribute to the development of MetS.

## Methods

### Study population

This community-based cross-sectional survey was conducted in Karachi, a metropolitan city in Pakistan, from February 2022 to August 2022. Karachi is one of the largest and most thickly populated cities in the world. The 2017 census report of Pakistan has reported Karachi’s population to be 14.9 million. It is the most ethnically diverse city in Pakistan and also serves as an economic hub of the country.

**Fig. 1:**
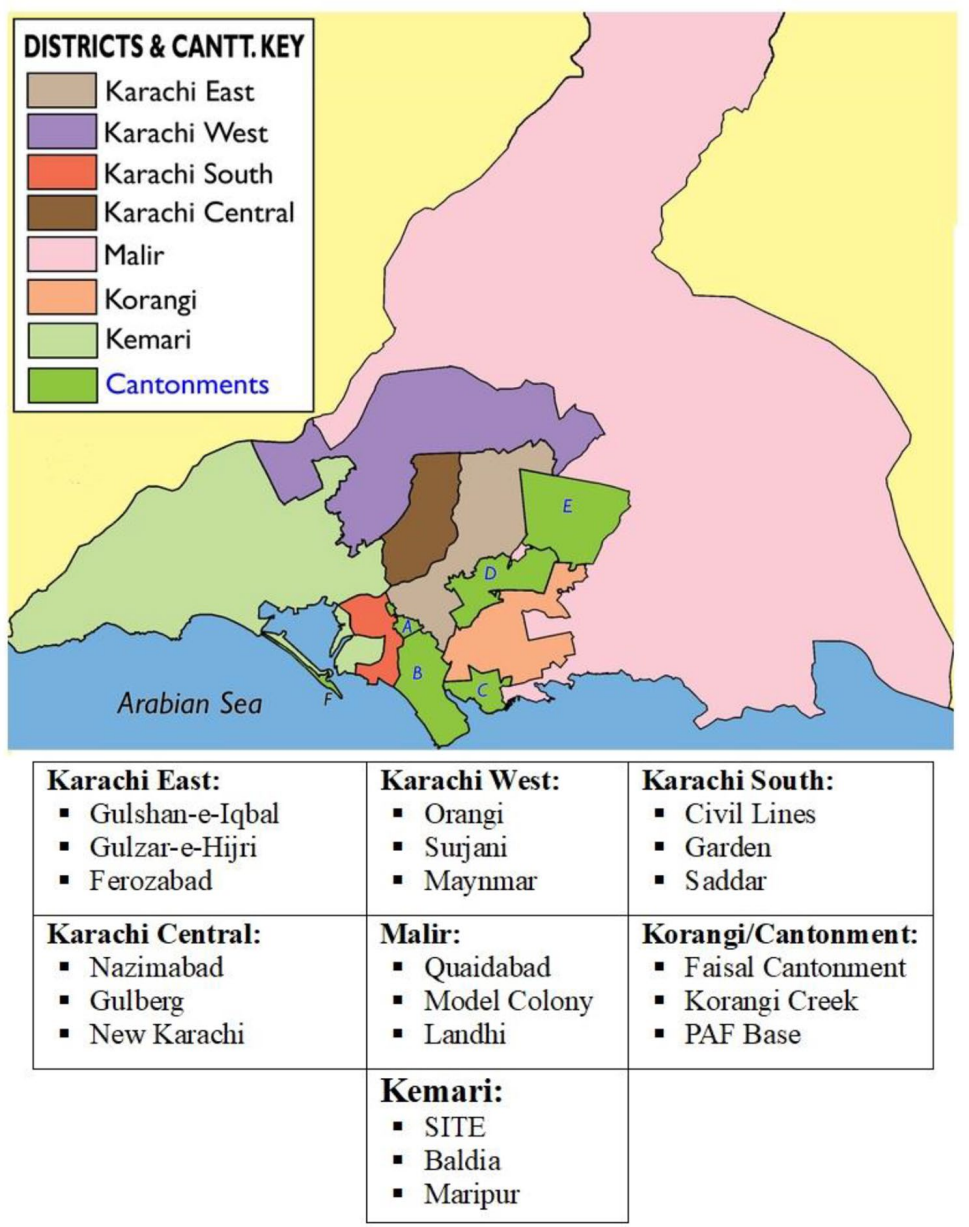
Geographic areas in Karachi, Pakistan selected for metabolic syndrome screening camps in the year 2022

A total of 21 screening camps were arranged in different areas of Karachi to cover the diversity of the population. These areas were selected based on the seven districts of Karachi. These were Karachi Central, Karachi East, Karachi West, Karachi South, Korangi, Malir, and Kemari. Three areas from each district were selected using non-probability consecutive sampling, and specialized medical camps for screening were arranged for apparently healthy individuals. The details of the areas where screening camps were placed are shown in Fig. 1.

The study focused on asymptomatic individuals who were healthy, free from physical disabilities, and had no known illnesses or regular medication usage. All such individuals aged 25–80 years of any gender were included. However, pregnant or lactating women and those with any missing component of MetS were excluded. Moreover, those individuals who perceived themselves as healthy but were underweight (< 18 kg/m^2^ BMI) or highly obese (> 40 kg/m^2^ BMI) were also excluded.

During this survey, a total of 2145 individuals underwent screening. However, 1080 individuals were excluded because they did not meet the criteria outlined in the pre-enrollment checklist for apparently healthy individuals. In addition, healthy individuals who were underweight or highly obese were also excluded. Consequently, 1065 individuals who met the criteria for being healthy were included in the study. The sample size estimation was performed using the prevalence of MetS according to the modified ATP III criteria in a research study conducted in Pakistan as 49%, a confidence interval of 95% CI, and a margin of error of 3%.

### Apparently healthy individuals

Apparently healthy individuals were defined as those who were not taking any medication regularly, had no physical disability, and perceived themselves as healthy based on the absence of any disease, signs, or symptoms. A pre-enrollment screening checklist was created to assess these individuals’ health status. The list included several components of an individual’s medical history, including pre-existing medical conditions (e.g., diabetes, hypertension, heart disease), past surgeries or hospitalizations, allergies (food, medication, environmental), and current medication usage.

### Metabolic syndrome

MetS was assessed using all the three internationally recognized definitions for MetS, i.e., NCEP-ATP III (2001), IDF (2005), and modified NCEP-ATP III (2005). The criteria for diagnosis of MetS by using three different definitions are mentioned in detail Table 1.

**Table 1:** Criteria for diagnosis of metabolic syndrome

	Metabolic Syndrome	Waist Circumference (WC)	Blood Pressure (BP)	Fasting Plasma Glucose	Triglyceride (TG)	HDL-C
NCEP-ATP III (2001)	*Presence of at least 3 RF*	For Males ≥ 102 cm, For Females ≥ 88 cm	SBP ≥ 130 mm/Hg and/or DBP ≥ 85 mm/Hg	≥ 110 mg/dL (≥ 6.1 mmol/L)	≥ 150 mg/dL (≥ 1.7 mmol/L)	For Males < 40 mg/dL (< 1.03 mmol/L), For Females < 50 mg/dL (< 1.29 mmol/L)
IDF (2005)	*WC with 2 or more other RF*	For Males ≥ 90 cm, For Females ≥ 80 cm	SBP ≥ 130 mm/Hg and/or DBP ≥ 85 mm/Hg or on treatment for hypertension	≥ 100 mg/dL (≥ 5.6 mmol/L) or previously diagnosed T2DM	≥ 150 mg/dL (≥ 1.7 mmol/L) or on treatment for TG	For Males < 40 mg/dL (< 1.03 mmol/L), For Females < 50 mg/dL (< 1.29 mmol/L) or on treatment for HDL-C
Modified NCEP-ATP III (2005)	*Presence of at least 3 RF*	For Males ≥ 90 cm, For Females ≥ 80 cm	SBP ≥ 130 mm/Hg and/or DBP ≥ 85 mm/Hg or on treatment for hypertension	≥ 100 mg/dL (≥ 5.6 mmol/L) or previously elevated glucose	≥ 150 mg/dL (≥ 1.7 mmol/L) or on treatment for TG	For Males < 40 mg/dL (< 1.03 mmol/L), For Females < 50 mg/dL (< 1.29 mmol/L) or on treatment for HDL-C

DBP: Diastolic blood pressure, HDL: High-Density Lipoprotein, NCEP-ATP III: National Cholesterol Education Program for Adult Treatment Panel, RF: Risk Factors, SBP: Systolic blood pressure

### Data collection procedure

A pre-structured questionnaire was used for the collection of the data. Detailed information was collected regarding the sociodemographic characteristics of the individuals, including age, gender, total monthly family income, religion, family structure, nature of the house, education, working status, breakfast skipping, physical activity (vigorous/moderate/low), chewing/addictive habits such as smoking, areca nut (betel nut) use, waterpipe smoking or chewing tobacco. Moreover, a family history of diabetes and hypertension was also noted. In addition, mental disorders were assessed by asking questions about anxiety during the past 12 months and suicidal ideation in the past 12 months. Body measurements like weight, height, BMI, and waist circumference (WC) were measured on the site of screening camps. WC was measured at the midpoint between the lower margin of the last palpable rib and the top of the iliac crest. In addition, clinical and laboratory parameters of the individuals, such as systolic blood pressure (SBP), diastolic blood pressure (DBP), fasting plasma glucose, High-Density Lipoprotein (HDL), and Triglycerides (TG), were noted. For laboratory investigation, two phlebotomists were arranged in each medical camp to collect the blood samples of those healthy individuals who visited the screening camp with at least 10 h of fasting. While a free coupon from Dow University Laboratory was distributed to those healthy individuals who came without fasting.

### Data analysis plan

Statistical analysis was performed using Statistical Package for Social Sciences (SPSS) version 24. Mean and standard deviation were reported for age, weight, height, BMI, HDL-C, TG, and WC. However, median and interquartile range (IQR) were calculated for total monthly household income. Frequency and percentages were calculated for qualitative variables, including the diagnosis of MetS, using different criteria and associated qualitative risk factors. Both univariate and multiple binary logistic regression was applied to determine the associated risk factors of MetS. Furthermore, each MetS component was evaluated separately for its association with predicting aspects. In the multivariable binary logistic regression those variables found statistically significant at *p*-values of 0.05 in the chi-square test were included as covariates. The sensitivity and specificity of NCEP-ATP III and modified NCEP-ATP III were also calculated using IDF as the gold standard. Moreover, Cohen’s Kappa statistics were also computed to see the level of agreement between different definitions. The *p*-value of ≤ 0.05 (2-sided) was considered significant.

## Results

### Baseline characteristics

A total of 1065 healthy individuals were screened. The mean (SD) age of the participants was 42.66 (12.18) years. There were 667 (62.6%) males. The participants’ mean weight, height, and BMI were 71.69 (13.39) kg, 163.71 (10.34) cm, and 26.78 (4.61) kg/m^2,^ respectively. The median of monthly household income in United States dollars was $177.47 (with an interquartile range of $92.28 to $234.26). Most participants were Muslims, i.e., 1034 (97.1%). In terms of family structure, it was observed that family structure observed that most of the participants were living in a nuclear family structure 492 (46.2%), followed by joint family 465 (43.7%), while 108 (10.1%) participants were living alone. Most of the study participants lived in concrete houses, i.e., 911 (85.5%), while 751 (70.5%) were working. The majority of the participants were married, i.e., 875 (82.2%) (Table S1).

### Physical and psychosocial characteristics of the participants

Most of the study participants reported low physical activity, i.e., 766 (71.9%). Most participants reported rarely or never skipping breakfast, i.e., 650 (61.0%). Non-smokers were predominantly higher, i.e., 841 (79.0%). Tobacco chewing was reported by 121 (11.4%), areca nut use by 166 (15.6%), and waterpipe smoking by 21 (2.0%) individuals. Family history of diabetes was observed in 540 (50.7%), while hypertension history in the family was observed in 634 (59.5%) individuals. Anxiety was reported by 402 (37.7%), whereas suicidal ideation was by 90 (8.5%) individuals (Table S1).

### Prevalence of components of MetS by various definitions

The prevalence of central obesity using IDF and modified NCEP-ATP III definitions were found to be 73.9% (95% CI: 71.1–76.5) in both definitions. Similarly, elevated plasma glucose level was similar in both IDF and modified NCEP-ATP III, i.e., 19.5% (95% CI 17.1–22.0). While elevated triglycerides prevalence, reduced HDL prevalence, and high blood pressure prevalence were found to be 19.6% (95% C.I 17.2–22.1), 49.9% (95% C.I 46.8–52.9), and 51.1% (95% C.I 48.0-54.1) respectively using all three definitions (Table 2).

### Prevalence of MetS by using various definitions

The prevalence of MetS based on IDF definition was found to be 32.2% (95% C.I 29–35), by NCEP ATP III 22.4% (95% C.I 19–25), while by modified NCEP-ATP III 33.9% (95% C.I 31–36) (Table 2).

Among individuals with < 30 years of age, the prevalence of MetS based on IDF definition was found to be 22.6% (95% C.I 17.1–28.9), by NCEP ATP III 13.5% (9.1–18.9), while by modified NCEP-ATP III 25.9% (95% C.I 20.1–32.5). Among individuals 30–50 years of age, the prevalence of MetS based on IDF definition was found to be 33.1% (95% C.I 29.4–37.0), by NCEP-ATP III 23.6% (20.2–27.1), while by modified NCEP-ATP III 34.3% (95% C.I 30.5–38.2). Among individuals with > 50 years of age, the prevalence of MetS based on IDF definition was found to be 38.0% (95% C.I 31.9–44.3), by NCEP ATP III 27.2% (95% C.I 21.8–33.2), while by modified NCEP-ATP III 40.0% (95% C.I 33.9–46.4). In male individuals, the prevalence of MetS based on IDF definition was found to be 29.8% (95% C.I 26.4–33.5), by NCEP ATP III 19.2% (16.3–22.4), while by modified NCEP-ATP III 32.2% (95% C.I 28.7–35.9). In female individuals, the prevalence of MetS based on IDF definition was found to be 36.2% (95% C.I 31.4–41.1), by NCEP ATP III 27.9% (23.5–32.6), while by modified NCEP-ATP III 36.9% (95% C.I 32.2–41.2) (Table S2).

Area-wise stratification revealed that the highest prevalence of undiagnosed MetS based on all three criteria was observed in Kemari Area, i.e., 18 (41.9%) through IDF and modified NCEP-ATP III definition, whereas 12 (27.9%) through NCEP-ATP III (Table S3).

### Comparison of MetS prevalence based on IDF with sociodemographic, physical, and psychosocial characteristics of the participants

Individuals with MetS had a significantly higher mean age (*p*-value < 0.001) and BMI (*p*-value ˂0.001) compared to individuals without MetS. Moreover, a significant association of MetS was observed with gender (*p*-value 0.032), current working (*p*-value 0.001), marital status (*p*-value 0.024), smoking status (*p*-value < 0.001), areca nut use (*p*-value 0.023), physical activity (*p*-value 0.037), and family history of hypertension (*p*-value 0.048) (Table 3).

### Comparison of MetS prevalence based on NCEP ATP III definition with sociodemographic, physical, and psychosocial characteristics of the participants

Individuals with MetS had a significantly higher mean age (*p*-value 0.001) and BMI (*p*-value < 0.001). Moreover, a significant association of MetS was observed with gender (*p*-value 0.001), religion (*p*-value 0.031), current working (*p*-value 0.001), marital status (*p*-value 0.048), smoking status (*p*-value < 0.001), and physical activity (*p*-value 0.002) (Table 3).

### Comparison of MetS prevalence based on modified NCEP ATP III definition with sociodemographic, physical, and psychosocial characteristics of the participants

Individuals with MetS had a significantly higher mean age (*p*-value 0.001) and BMI (*p*-value < 0.001). Moreover, a significant association of MetS was observed with current working (*p*-value 0.002), smoking status (*p*-value < 0.001), areca nut use (*p*-value 0.039), physical activity (*p*-value 0.002), and family history of hypertension (*p*-value 0.041) (Table 3).

### Regression analysis of variables associated with undiagnosed MetS

The findings of the multiple binary logistic analysis revealed that after adjustment of other covariates, based on IDF definition, the odds of prevalence of undiagnosed MetS was significantly higher in individuals with increased BMI (ORadj 1.13, 95% CI 1.09–1.17), areca nut use (ORadj 1.71, 95% CI 1.19–2.47), and current smoking (ORadj 2.54, 95% CI 1.73–3.73). Based on NCEP-ATP III criteria, the odds of prevalence of undiagnosed MetS were significantly higher in female individuals (ORadj 1.85, 95% CI 1.24–2.78), increased BMI (ORadj 1.15, 95% CI 1.11–1.19), current smoking (ORadj 3.77, 95% CI 2.47–5.75), and low physical activity (ORadj 1.56, 95% CI 1.08–2.26). Based on the modified NCEP ATP III definition, the odds of prevalence of undiagnosed MetS were significantly higher among individuals with increased BMI (ORadj 1.12, 95% CI 1.08–1.15), areca nut use (ORadj 1.58, 95% CI 1.10–2.72), current smoking (ORadj 2.24, 95% CI 1.55–3.21), and low physical activity (ORadj 1.36, 95% CI 1.01–1.84). Yet, the odds of prevalence of undiagnosed MetS were significantly lower in individuals currently working (ORadj 0.70, 95% CI 0.52–0.95) (Table 4).

### Sensitivity and specificity analysis

Kappa statistics revealed a substantial agreement between IDF and NCEP-ATP III definition (k = 0.645, *p*-value < 0.001). A strong agreement was observed between IDF and modified NCEP-ATP III guidelines (k = 0.960, *p*-value < 0.001). Modified NCEP-ATP III was more sensitive than NCEP-ATP III, i.e., 94.7% and 62.7%, respectively (Table 5). Age and gender-wise stratification revealed similar findings (Table S4).

**Table 2:** Prevalence of components of undiagnosed metabolic syndrome among apparently healthy individuals living in Karachi, Pakistan in the year 2022

	IDF	NCEP ATP III	Modified NCEP-ATP III
Metabolic Abnormalities	% (95% CI)	% (95% CI)	% (95% CI)
Central Obesity	73.9 (71.1–76.5)	38.9 (35.9–41.8)	73.9 (71.1–76.5)
Elevated Fasting Plasma Glucose	19.5 (17.1–22.0)	11.6 (9.7–13.7)	19.5 (17.1–22.0)
Elevated Triglyceride	19.6 (17.2–22.1)	19.6 (17.2–22.1)	19.6 (17.2–22.1)
Reduced HDL	49.9 (46.8–52.9)	49.9 (46.8–52.9)	49.9 (46.8–52.9)
High Blood Pressure	51.1 (48.0-54.1)	51.1 (48.0-54.1)	51.1 (48.0-54.1)
Metabolic Syndrome	32.2 (29.0–35.0)	22.4 (19.0–25.0)	33.9 (31.0–36.0)

CI: Confidence Interval, HDL: High Density Lipoprotein-Cholesterol, IDF: International Diabetes Federation, NCEP-ATP: National Cholesterol Education Programme Adult Treatment Panel

**Table 3:** Comparison of demographic and clinical features of apparently healthy and undiagnosed metabolic syndrome individuals living in Karachi, Pakistan in the year 2022

Variables	IDF			NCEP-ATP III			Modified NCEP-ATP III		
	MetS	Non-MetS	*p*-value	MetS	Non-MetS	*p*-value	MetS	Non-MetS	*p*-value
	n (%)	n (%)		n (%)	n (%)		n (%)	n (%)	
*N*	*343*	*722*		239	826		362	703	
Age (years), mean ± SD	44.54 ± 11.60	41.76 ± 12.35	< 0.001	45.01 ± 11.01	41.98 ± 12.42	0.001	45.01 ± 11.01	41.98 ± 12.43	0.001
Male, n (%)	199 (29.8)	468 (70.2)	0.032	128 (19.2)	539 (80.8)	0.001	215 (32.2)	452 (67.8)	0.117
Body Mass Index, kg/m2	28.64 ± 4.16	25.90 ± 4.54	< 0.001	29.28 ± 4.47	26.05 ± 4.39	< 0.001	29.28 ± 4.47	26.05 ± 4.39	< 0.001
Muslim, n (%)	336 (32.5)	698 (67.5)	0.454	238 (23.0)	796 (77.0)	0.031	355 (34.3)	679 (65.7)	0.355
Family Structure									
Alone, n (%)	26 (24.1)	82 (75.9)	0.087	15 (13.9)	93 (86.1)	0.067	27 (25.0)	81 (75.0)	0.075
Nuclear, n (%)	171 (34.8)	321 (65.2)		119 (24.2)	373 (75.8)		179 (36.4)	313 (63.6)	
Joint, n (%)	146 (31.4)	319 (68.6)		105 (22.6)	360 (77.4)		156 (33.5)	309 (66.5)	
Concrete house (Pukka), n (%)	300 (32.9)	611 (67.1)	0.219	204 (22.4)	707 (77.6)	0.927	316 (34.7)	595 (65.3)	0.243
Education									
Graduate or above, n (%)	151 (32.6)	312 (67.4)	0.891	100 (21.6)	363 (78.4)	0.195	159 (34.3)	304 (65.7)	0.987
Matriculation or intermediate, n (%)	110 (32.1)	233 (67.9)		72 (21.0)	271 (79.0)		114 (33.2)	229 (66.8)	
Primary or secondary, n (%)	39 (29.5)	93 (70.5)		29 (22.0)	103 (78.0)		45 (34.1)	87 (65.9)	
No Schooling, n (%)	43 (33.9)	84 (66.1)		38 (29.9)	89 (70.1)		44 (34.6)	83 (65.4)	
Currently working, n (%)	219 (29.2)	532 (70.8)	0.001	147 (19.6)	604 (80.4)	0.001	233 (31.0)	518 (69.0)	0.002
Married, n (%)	295 (33.7)	580 (66.3)	0.024	205 (23.4)	670 (76.6)	0.048	309 (35.3)	566 (64.7)	0.097
Smoker									
Current Smoker, n (%)	77 (47.5)	85 (52.5)	< 0.001	62 (38.3)	100 (61.7)	< 0.001	79 (48.8)	83 (51.2)	< 0.001
Ex-Smoker, n (%)	25 (40.3)	37 (59.7)		14 (22.6)	48 (77.4)		26 (41.9)	36 (58.1)	
Non-Smoker, n (%)	241 (28.7)	600 (71.3)		163 (19.4)	678 (80.6)		257 (30.6)	584 (69.4)	
Waterpipe Smoker, n (%)	5 (23.8)	16 (76.2)	0.406	6 (28.6)	15 (71.4)	0.496	7 (33.3)	14 (66.7)	0.949
Area Nut Use, n (%)	66 (39.8)	100 (60.2)	0.023	44 (26.5)	122 (73.5)	0.172	68 (41.0)	98 (59.0)	0.039
Chew Tobacco, n (%)	44 (36.4)	77 (63.6)	0.299	24 (19.8)	97 (80.2)	0.465	46 (38.0)	75 (62.0)	0.321
Breakfast Skipping									
Usually/Often, n (%)	96 (32.4)	200 (67.6)	0.214	69 (23.3)	227 (76.7)	0.539	102 (34.5)	194 (65.5)	0.218
Sometimes, n (%)	30 (25.2)	89 (74.8)		22 (18.5)	97 (81.5)		32 (26.9)	87 (73.1)	
Rarely/Never, n (%)	217 (33.4)	433 (66.6)		148 (22.8)	502 (77.2)		228 (35.1)	422 (64.9)	
Physical Activity									
Low, n (%)	261 (34.1)	505 (65.9)	0.037	191 (24.9)	575 (75.1)	0.002	278 (36.3)	488 (63.7)	0.011
Moderate/Vigorous, n (%)	82 (27.4)	217 (72.6)		48 (16.1)	251 (83.9)		84 (28.1)	215 (71.9)	
Family History of Diabetes, n (%)	172 (31.9)	368 (68.1)	0.802	116 (21.5)	424 (78.5)	0.446	183 (33.9)	357 (66.1)	0.943
Family history of Hypertension, n (%)	219 (34.5)	415 (65.5)	0.048	153 (24.1)	481 (75.9)	0.109	231 (36.4)	403 (63.6)	0.041
Anxiety, n (%)	139 (34.6)	263 (65.4)	0.197	94 (23.4)	308 (76.6)	0.566	146 (36.3)	256 (63.7)	0.212
Suicidal Ideation, n (%)	31 (34.4)	59 (65.6)	0.635	23 (25.6)	67 (74.4)	0.459	31 (34.4)	59 (65.6)	0.924

Data were presented as number (%) for categorical variables and mean ± SD

*P-value: for continuous variables, estimates were based on an Independent t-test; for categorical variables, estimates were based on the Pearson’s Chi-square test

**Table 4:** Logistic regression analysis of variables associated with undiagnosed metabolic syndrome among individuals living in Karachi, Pakistan in the year 2022

	**MetS using ID**F			
**Variables**	**Univariable Analysis**		**Multivariable Analysis** ^α^	
	**ORunadj (95% CI)**	***p*** **-value**	**ORadj (95% CI)**	***p*** **-value**
Age (years), mean ± SD	1.02 (1.01–1.03)	0.001	1.01 (0.99–1.01)	0.068
Female vs. Male, n (%)	1.33 (1.03–1.74)	0.032	1.32 (0.92–1.87)	0.131
Body Mass Index, kg/m2	1.14 (1.11–1.17)	< 0.001	1.13 (1.09–1.17)	< 0.001
Area Nut Use, n (%)	1.48 (1.05–2.09)	0.024	1.71 (1.19–2.47)	0.004
Currently working, n (%)	0.63 (0.48–0.83)	0.001	0.80 (0.56–1.15)	0.223
Married, n (%)	1.51 (1.05–2.15)	0.024	1.17 (0.79–1.73)	0.431
Current vs. Ex/Non-Smoker	2.17 (1.54–3.05)	< 0.001	2.54 (1.73–3.73)	< 0.001
Low vs. Moderate/Vigorous Physical Activity, n (%)	1.37 (1.02–1.84)	0.037	1.25 (0.91–1.71)	0.165
Family history of Hypertension, n (%)	1.31 (1.01–1.70)	0.048	1.07 (0.80–1.43)	0.636
	**MetS using NCEP-ATP III**			
**Variables**	**Univariable Analysis**		**Multivariable Analysis** ^**β**^	
	**ORunadj (95% CI)**	***p*** **-value**	**ORadj (95% CI)**	***p*** **-value**
Age (years), mean ± SD	1.02 (1.01–1.03)	0.001	1.01 (1.00-1.03)	0.052
Female vs. Male, n (%)	1.63 (1.22–2.18)	0.001	1.85 (1.24–2.78)	0.003
Body Mass Index, kg/m2	1.17 (1.13–1.20)	< 0.001	1.15 (1.11–1.19)	< 0.001
Currently working, n (%)	0.58 (0.43–0.79)	0.001	0.84 (0.57–1.25)	0.389
Current vs. Ex/Non-Smoker	2.54 (1.78–3.63)	< 0.001	3.77 (2.47–5.75)	< 0.001
Low vs. Moderate/Vigorous Physical Activity, n (%)	1.74 (1.22–2.46)	0.002	1.56 (1.08–2.26)	0.018
	**MetS using Modified NCEP-ATP III**			
**Variables**	**Univariable Analysis**		**Multivariable Analysis** ^**¥**^	
	**ORunadj (95% CI)**	***p*** **-value**	**ORadj (95% CI)**	***p*** **-value**
Age (years), mean ± SD	1.02 (1.01–1.03)	0.001	1.01 (0.99–1.02)	0.139
Body Mass Index, kg/m2	1.16 (1.13–1.20)	< 0.001	1.12 (1.08–1.15)	< 0.001
Area Nut Use, n (%)	1.43 (1.02–2.01)	0.040	1.58 (1.10–2.72)	0.012
Currently working, n (%)	0.58 (0.43–0.79)	0.001	0.70 (0.52–0.95)	0.023
Current vs. Ex/Non-Smoker	2.08 (1.48–2.93)	< 0.001	2.24 (1.55–3.21)	< 0.001
Low vs. Moderate/Vigorous Physical Activity, n (%)	1.74 (1.23–2.46)	0.002	1.36 (1.01–1.84)	0.048
Family history of Hypertension, n (%)	1.31 (1.01–1.72)	0.041	1.12 (0.85–1.48)	0.412

ORunadj: Unadjusted Odds Ratio, ORadj: Adjusted Odds Ratio, CI: Confidence Interval, IDF: International Diabetes Federation, NCEP-ATP: National Cholesterol Education Programme Adult Treatment Panel, OR: Odds Ratio

^α^Adjusted for age, gender, BMI, areca nut use, current working status, marital status, smoking, physical activity, and family history of hypertension

^β^Adjusted for age, gender, BMI, current working status, smoking, and physical activity

^¥^Adjusted for age, BMI, areca nut use, current working status, smoking, physical activity, and family history of hypertension

**Table 5:** Sensitivity, specificity, and level of agreement of NCEP ATP III and modified NCEP ATP III for MetS using IDF as standard definition

Definition	IDF			
	Sensitivity	Specificity	Cohen’s Kappa Index	*p*-value
	% (95% CI)	% (95% CI)		
NCEP ATP III	62.7 (57.3–67.8)	96.7 (95.1–97.9)	0.645	< 0.001
Modified NCEP ATP III	94.7 (91.9–96.8)	100 (99.4–100)	0.960	< 0.001

## Discussion

The current study’s findings have yielded significant results regarding the prevalence and associated risk factors of undiagnosed MetS among apparently healthy individuals. According to the study findings, one-third of the apparently healthy individuals had undiagnosed MetS based on IDF and modified NCEP-ATP III definitions. Though the prevalence of undiagnosed MetS as defined by NCEP-ATP III guidelines was one-fourth which is a little bit lower than IDF and modified NCEP-ATP III, its prevalence is still higher as all these individuals perceive themselves as healthy and delay in the diagnoses could have led to fatal consequences. Though, as per the current study findings, both modified NCEP-ATP III and IDF reported somewhat similar results, the IDF criteria are a recommended choice for their global applicability and relevance to diverse population. The main differences lie in the abdominal obesity criterion and the handling of fasting glucose levels. The modified NCEP ATP III criteria allow for varying WC cutoffs based on gender and ethnicity, while the IDF criteria have uniform cutoffs globally. Moreover, the IDF criteria explicitly mention previously diagnosed type 2 diabetes for the fasting glucose component, whereas the modified NCEP ATP III criteria use elevated fasting glucose levels or diabetes medication. Given the increasing prevalence of diabetes in Pakistan, explicitly mention previously diagnosed type 2 diabetes for the fasting glucose component can help identify individuals at higher risk for diabetes-related complications.

The fact that a significant portion of individuals with undiagnosed MetS perceived themselves as healthy raises concerns about the potential consequences of delayed diagnosis. MetS can lead to severe and even fatal outcomes if left undetected and untreated. The diagnosis delay could have resulted in missed opportunities for early interventions and lifestyle modifications that could have mitigated the risk factors associated with MetS. Similar findings were reported in studies carried out in other Asian countries, particularly neighboring countries of Pakistan [19–23]. However, a higher prevalence is reported in a recently published study from Iran in which the prevalence of MetS in the general population was 38.3% as per the IDF definition and 43.5% as per the NCEP-ATP III definition [20]. The findings of various recently published meta-analyses from Asian countries also reported the prevalence of MetS in one-third of the general population [22, 24–28]. Most of the recently published studies conducted in Pakistan have said that the undiagnosed prevalence of MetS ranged from 20 to 39.23% [29–31]. However, Malik et al. reported 6.1%, and Ahmed et al. reported 55.4% prevalence in their study [12, 32]. A possible reason for the extremely low prevalence in Malik et al. may be because the study was conducted in young individuals (18–24 years) only. Younger age individuals also had a relatively lower risk of MetS as evidenced by the current study. While the possible reason for the higher risk in Ahmed et al. study is the inclusion of participants with an increased risk of diabetes. While we could not provide a detailed breakdown of the age-specific proportion of MetS in our study, our findings suggest that age is a significant risk factor for the development of MetS. These findings highlight the importance of early screening and interventions for MetS, especially in older individuals, to prevent or delay the onset of related health complications.

The current study findings from all three MetS definitions have revealed a higher prevalence of undiagnosed MetS in the Kemari Area of Karachi, followed by the West and Korangi areas. Although some studies have reported the prevalence of MetS among the healthy population of Karachi city [16, 32–34], none of the studies has reported area-wise prevalence. Therefore, this study is the first of its kind from Karachi city that has reported area-wise prevalence, so comparison is impossible. However, one study that has reported ethnic-wise distribution of MetS has reported a higher prevalence of Mets among Pashtuns while lower in Urdu Speaking (Indian Immigrants) population [33].

According to the current study findings, increased waist circumference and hypertension were the two essential components of MetS that were predominantly higher than other components of MetS. In particular, the IDF and modified NCEP-ATP III criteria have revealed that three-fourths of the participants had increased waist circumference in the current study. This higher prevalence of central obesity is also evident in increased BMI levels in the current study. Moreover, several previously published studies have also reported waist circumference as an important anthropometric parameter for predicting the risk of MetS [35–37].

Though higher education is reported as one of the important factors in the development of MetS in one of the previous studies from Karachi, however, no considerable role of educational status or socioeconomic status was observed in the current study. This finding is alarming and indicates the need for prompt action in creating awareness and controlling the risk of the increasing prevalence of MetS in residents of Karachi. The prevalence of MetS in Karachi can be attributed to the unhealthy diet, lack of physical activity, chronic stress, and smoking habits of the people living in the city [38].

In the current study, a considerably higher proportion of MetS was observed in apparently healthy individuals who had low physical activity, chewing areca nut, and smoking. These factors can be modified through lifestyle interventions, education, and awareness campaigns to reduce the prevalence of MetS in Karachi.

There are certain limitations in the current study. Firstly, the study did not cover the peri-urban areas as these areas are outside the city limits; hence these areas were excluded from the study. However, peri-urban areas are also thickly populated. A similar study is also needed to screen apparently healthy individuals in these areas. Secondly, some of the potential participants that showed up to the screening camps were not fasting either due to the lack of information or due to negligence; hence their data had to be excluded from the study as lipid profiles and fasting plasma sugar requires the fasting state of the individuals. To cope with this problem, free coupons for laboratory tests were widely distributed amongst the potential candidates who came without fasting. However, some did not avail themselves of the free coupons, and their laboratory parameters were unavailable. Consequently, the MetS status of healthy potential candidates was missed. One of the problems that were noticed in the current study is that a lot of the participants that were given the free lab coupons used them for their family members; hence the lab data did not match the parameters that the researcher took in the screening camp and therefore had to be excluded. Future researchers should adopt a stricter adherence technique to ensure maximum compliance and correct use of free health coupons.

Despite these limitations, the study is of significant importance as this was a large community-based study that was inclusive of people from all significant socioeconomic, educational, cultural, and religious backgrounds. As Karachi is an economic hub and a metropolitan city, it is easy to access a wide variety of people to ensure inclusivity in the study and that people from different backgrounds are included. This study is one of the first studies from Karachi city that have extensively reported the prevalence of MetS among apparently healthy individuals. The current study covered all parts of the metropolitan area from north, south, west, east, and central Karachi, which is not reported in previously published studies. Furthermore, the study focused mainly on apparently healthy individuals with no underlying conditions; therefore, this study excluded underweight (< 18 BMI) and extremely obese (BMI > 40) individuals. This was very important as all the chosen participants had no known co-morbidities and were leading an everyday healthy life. Lastly, the study has reported MetS using all internationally recognized definitions of MetS. Thus, a comparison of the prevalence of MetS using all definitions is reported.

The study’s findings revealed a considerably higher number of apparently healthy individuals with MetS and have essential implications in real life. The study highlights the importance of implementing screening programs for healthy individuals to detect MetS. It allows for timely intervention and management to prevent the progression of the condition and reduce the risk of associated health complications. Furthermore, identifying risk factors such as elevated BMI, smoking, areca nut chewing, and low physical activity among apparently healthy individuals has brought attention to those at risk. This recognition allows healthcare providers to provide targeted interventions and customized lifestyle modifications to these individuals, ultimately leading to improved health outcomes.

Further studies are recommended to screen individuals with high-risk factors such as high BMI, smoking, areca nut chewers, and low physical activity, as reported in this study. Moreover, a study with a long-term follow-up period is recommended to track changes in participants’ health status and identify risk factors associated with the development of MetS. Longitudinal studies can also help to establish the natural history of MetS and its related complications, such as cardiovascular disease and type 2 diabetes. By following individuals over time, researchers can better understand the long-term health outcomes associated with MetS and identify factors that may influence disease progression and treatment response. In addition to traditional risk factors, biomarkers such as inflammatory markers and adipokines have been associated with MetS. Future research should evaluate the utility of these biomarkers in screening for MetS. Lastly, screening for MetS can be costly, and evaluating the cost-effectiveness of different screening strategies is important. Future research should assess the cost-effectiveness of various screening protocols and determine the most efficient approach for identifying individuals with MetS.

## Conclusion

The study has reported undiagnosed MetS in one-third of the healthy individuals living in different areas of Karachi, using IDF, NCEP-ATP III, and modified NCEP-ATP III criteria. In particular, individuals with increased BMI, who are currently smoking, chew areca nuts and have limited physical activity are vulnerable populations and at higher risk of MetS. As all these are modifiable risk factors, the risk of MetS and its components could be prevented after making some efforts in daily life activities. Also, there is a need to develop strategies to increase awareness among apparently healthy individuals to get themselves screened for MetS and its components to timely diagnose the silent diseases such as MetS that slowly progress in the body and potentially cause negative health consequences. Though, as per the current study findings, both modified NCEP-ATP III and IDF reported somewhat similar results, the IDF criteria are a recommended choice for their global applicability and relevance to diverse population.

### Electronic supplementary material

Below is the link to the electronic supplementary material.

Supplementary Material 1

## Data Availability

The raw data supporting the conclusions of this article will be made available by the authors, without undue reservation.
