# Supplementary material for: Prevalence of undiagnosed metabolic syndrome using three different definitions and identifying associated risk factors among apparently healthy adults in Karachi, Pakistan: a cross-sectional survey in the year 2022
Source: Arch Public Health. 2024 Feb 20;82:22. doi: 10.1186/s13690-024-01250-3 (PMC10877913; doi:10.1186/s13690-024-01250-3)
Supplement: Supplementary file 1 — Supplementary Material 1 [file 13690_2024_1250_MOESM1_ESM.docx]

| **Supplementary Table 1: Baseline, physical and psychosocial characteristics of the study participants (n=1065)** | | |
| --- | --- | --- |
|  | **n** | **%** |
| **Age, years** | 42.66 ±12.18 | |
| **Gender** |  |  |
| Male | 667 | 62.6 |
| Female | 398 | 37.4 |
| **Weight, kg** | 71.69 ±13.39 | |
| **Height, cm** | 163.71 ±10.34 | |
| **BMI, kg/m2** | 26.78 ±4.61 | |
| **Monthly income, rupees** | 50,000 (26,000-66,000) | |
| **Religion** | | |
| Muslim | 1034 | 97.1 |
| Christian | 16 | 1.5 |
| Hindu | 15 | 1.4 |
| **Family Structure** | | |
| Alone | 108 | 10.1 |
| Nuclear | 492 | 46.2 |
| Joint | 465 | 43.7 |
| **Nature of House** | | |
| Non-Concreate (Kuchha) | 154 | 14.5 |
| Concreate (Pukka) | 911 | 85.5 |
| **Education** | | |
| Graduate or above | 463 | 43.5 |
| Matriculation or intermediate | 343 | 32.2 |
| Primary or secondary | 132 | 12.4 |
| No Schooling | 127 | 11.9 |
| **Work Currently** | 751 | 70.5 |
| **Marital Status** | | |
| Single | 152 | 14.3 |
| Married | 875 | 82.2 |
| Widow/Divorced | 38 | 3.6 |
| **Breakfast Skipping** | | |
| Usually/Often | 296 | 27.8 |
| Sometimes | 119 | 11.2 |
| Rarely/Never | 650 | 61.0 |
| **Physical Activity** | | |
| Low | 766 | 71.9 |
| Moderate | 187 | 17.6 |
| Vigorous | 112 | 10.5 |
| **Smoker** | | |
| Current Smoker | 162 | 15.2 |
| Ex-Smoker | 62 | 5.8 |
| Non-Smoker | 841 | 79.0 |
| **Waterpipe Smoker** | 21 | 2.0 |
| **Area Nut Use** | 166 | 15.6 |
| **Chew Tobacco** | 121 | 11.4 |
| **Family History of Diabetes** | 540 | 50.7 |
| **Family history of Hypertension** | 634 | 59.5 |
| **Anxiety** | 402 | 37.7 |
| **Suicidal Ideation** | 90 | 8.5 |

| **Supplementary Table 2: Prevalence of components of undiagnosed metabolic syndrome stratified by age and gender** | | | |
| --- | --- | --- | --- |
| **Metabolic Abnormalities** | **IDF** | **NCEP ATP III** | **Modified NCEP-ATP III** |
|  | **% (95% CI)** | **% (95% CI)** | **% (95% CI)** |
| **Age <30 years (n=208)** | | | |
| Central Obesity | 70.7 (63.9-76.7) | 38.9 (32.3-45.9) | 70.7 (63.9-76.7) |
| Elevated Fasting Plasma Glucose | 15.4 (10.7-21.0) | 10.1 (6.4-15.0) | 15.4 (10.7-21.0) |
| Elevated Triglyceride | 18.3 (13.3-24.2) | 18.3 (13.3-24.2) | 18.3 (13.3-24.2) |
| Reduced HDL | 65.9 (58.9-72.3) | 65.9 (58.9-72.3) | 65.9 (58.9-72.3) |
| High Blood Pressure | 42.8 (35.9-49.8) | 42.8 (35.9-49.8) | 42.8 (35.9-49.8) |
| Metabolic Syndrome | 22.6 (17.1-28.9) | 13.5 (9.1-18.9) | 25.9 (20.1-32.5) |
| **Age 30-50 years (n=607)** | | | |
| Central Obesity | 74.3 (70.6-77.7) | 36.7 (32.9-40.7) | 74.3 (70.6-77.7) |
| Elevated Fasting Plasma Glucose | 17.6 (14.7-20.9) | 17.6 (14.7-20.9) | 17.6 (14.7-20.9) |
| Elevated Triglyceride | 19.9 (16.8-23.3) | 19.9 (16.8-23.3) | 19.9 (16.8-23.3) |
| Reduced HDL | 47.3 (43.2-51.3) | 47.3 (43.2-51.3) | 47.3 (43.2-51.3) |
| High Blood Pressure | 50.1 (46.5-54.6) | 50.1 (46.5-54.6) | 50.1 (46.5-54.6) |
| Metabolic Syndrome | 33.1 (29.4-37.0) | 23.6 (20.2-27.1) | 34.3 (30.5-38.2) |
| **Age >50 years (n=250)** | | | |
| Central Obesity | 75.6 (69.8-80.8) | 44.0 (37.8-50.4) | 75.6 (69.8-80.8) |
| Elevated Fasting Plasma Glucose | 27.6 (22.2-33.6) | 17.6 (13.1-22.9) | 27.6 (22.2-33.6) |
| Elevated Triglyceride | 20.0 (15.2-25.5) | 20.0 (15.2-25.5) | 20.0 (15.2-25.5) |
| Reduced HDL | 42.8 (36.6-49.2) | 42.8 (36.6-49.2) | 42.8 (36.6-49.2) |
| High Blood Pressure | 59.2 (52.8-65.4) | 59.2 (52.8-65.4) | 59.2 (52.8-65.4) |
| Metabolic Syndrome | 38.0 (31.9-44.3) | 27.2 (21.8-33.2) | 40.0 (33.9-46.4) |
| **Male Gender (n=667)** | | | |
| Central Obesity | 73.9 (70.4-77.2) | 44.9 (41.2-48.8) | 73.9 (70.4-77.2) |
| Elevated Fasting Plasma Glucose | 18.7 (15.6-21.9) | 11.4 (9.1-14.1) | 18.7 (15.6-21.9) |
| Elevated Triglyceride | 24.6 (21.4-28.0) | 24.6 (21.4-28.0) | 24.6 (21.4-28.0) |
| Reduced HDL | 56.8 (52.9-60.6) | 56.8 (52.9-60.6) | 56.8 (52.9-60.6) |
| High Blood Pressure | 54.7 (50.9-58.6) | 54.7 (50.9-58.6) | 54.7 (50.9-58.6) |
| Metabolic Syndrome | 29.8 (26.4-33.5) | 19.2 (16.3-22.4) | 32.2 (28.7-35.9) |
| **Female Gender (n=398)** | | | |
| Central Obesity | 73.4 (69.2-78.1) | 28.6 (24.3-33.4) | 73.4 (69.2-78.1) |
| Elevated Fasting Plasma Glucose | 20.9 (16.9-25.2) | 12.1 (9.0-15.7) | 20.9 (16.9-25.2) |
| Elevated Triglyceride | 11.3 (8.4-14.8) | 11.3 (8.4-14.8) | 11.3 (8.4-14.8) |
| Reduced HDL | 38.2 (33.4-43.2) | 38.2 (33.4-43.2) | 38.2 (33.4-43.2) |
| High Blood Pressure | 44.9 (40.1-50.0) | 44.9 (40.1-50.0) | 44.9 (40.1-50.0) |
| Metabolic Syndrome | 36.2 (31.4-41.1) | 27.9 (23.5-32.6) | 36.9 (32.2-41.2) |
| CI: Confidence Interval, HDL: High Density Lipoprotein-Cholesterol, IDF: International Diabetes Federation, NCEP-ATP: National Cholesterol Education Programme Adult Treatment Panel | | | |

| **Supplementary Table 3: Area-wise prevalence of MetS using different definitions** | | | | |
| --- | --- | --- | --- | --- |
| **Area** | **n** | **MetS using IDF** | **MetS using NCEP ATP III** | **MetS using Modified NCEP ATP III** |
|  |  | **% (95% CI)** | **% (95% CI)** | **% (95% CI)** |
| **Total (n=1065)** | | | | |
| South | 78 | 21.8 (13.2-32.5) | 19.2 (11.2-29.7) | 23.1 (14.3-34.0) |
| West | 102 | 35.3 (26.1-45.4) | 27.5 (19.1-37.2) | 38.2 (28.8-48.4) |
| East | 234 | 30.3 (24.5-36.7) | 21.8 (16.7-27.6) | 32.1 (26.1-38.4) |
| Central | 276 | 32.6 (27.1-38.5) | 21.4 (16.7-26.7) | 35.5 (29.9-41.5) |
| Korangi | 111 | 36.0 (27.1-45.7) | 27.0 (19.0-36.3) | 36.9 (27.9-46.6) |
| Malir | 221 | 32.1 (25.9-38.3) | 19.9 (14.8-25.8) | 33.0 (26.9-39.7) |
| Kemari | 43 | 41.9 (27.0-57.9) | 27.9 (15.3-43.7) | 41.9 (27.0-57.9) |
| **Age <30 years (n=208)** | | | | |
| South | 23 | 26.1 (10.2-48.4) | 17.4 (4.9-38.7) | 30.4 (13.2-52.9) |
| West | 17 | 11.8 (1.5-36.4) | 11.8 (1.5-36.4) | 17.6 (3.8-43.4) |
| East | 55 | 27.3 (16.1-40.9) | 10.9 (4.1-22.3) | 29.1 (17.6-42.9) |
| Central | 56 | 17.9 (8.9-30.4) | 8.9 (2.9-19.6) | 23.2 (12.9-36.4) |
| Korangi | 14 | 35.7 (12.8-64.9) | 35.7 (12.8-64.9) | 42.9 (17.7-71.1) |
| Malir | 30 | 16.7 (5.6-34.7) | 10.0 (2.1-26.5) | 16.7 (5.6-34.7) |
| Kemari | 13 | 30.8 (9.1-61.4) | 23.1 (5.0-53.8) | 30.8 (9.1-61.4) |
| **Age 30-50 years (n=607)** | | | | |
| South | 39 | 20.5 (9.3-36.5) | 15.4 (5.9-30.5) | 20.5 (9.3-36.5) |
| West | 68 | 36.7 (25.4-49.3) | 26.5 (16.5-38.6) | 39.7 (28.0-52.3) |
| East | 137 | 28.5 (21.1-36.8) | 25.6 (18.5-33.7) | 29.9 (22.4-38.3) |
| Central | 159 | 38.4 (30.8-46.4) | 25.2 (18.6-32.6) | 39.6 (31.9-47.7) |
| Korangi | 51 | 27.5 (15.9-41.7) | 19.6 (9.8-33.1) | 27.5 (15.9-41.7) |
| Malir | 129 | 33.3 (25.3-42.2) | 21.7 (14.9-29.8) | 34.1 (25.9-42.9) |
| Kemari | 24 | 45.8 (25.6-67.2) | 25.0 (9.8-46.7) | 45.8 (25.6-67.2) |
| **Age >50 years (n=250)** | | | | |
| South | 16 | 18.8 (4.1-45.7) | 31.3 (11.0-58.7) | 18.8 (4.1-45.7) |
| West | 17 | 52.9 (27.8-77.0) | 47.1 (22.9-72.2) | 52.9 (27.8-77.0) |
| East | 42 | 40.5 (25.6-56.7) | 23.8 (12.1-39.5) | 42.9 (27.7-59.0) |
| Central | 61 | 31.1 (19.9-44.3) | 22.9 (13.2-35.5) | 36.1 (24.2-49.4) |
| Korangi | 46 | 45.7 (30.9-60.9) | 32.6 (19.5-48.0) | 45.7 (30.9-60.9) |
| Malir | 62 | 37.1 (25.2-50.3) | 20.9 (11.7-33.2) | 38.7 (26.6-51.9) |
| Kemari | 6 | 50.0 (11.8-88.2) | 50.0 (11.8-88.2) | 50.0 (11.8-88.2) |
| **Male gender (n=667)** | | | | |
| South | 59 | 18.6 (9.7-30.9) | 16.9 (8.4-28.9) | 20.3 (10.9-32.8) |
| West | 84 | 38.1 (27.7-49.3) | 28.6 (19.2-39.5) | 41.7 (31.0-52.9) |
| East | 130 | 29.2 (21.6-37.9) | 20.8 (14.2-28.8) | 32.3 (24.4-41.1) |
| Central | 188 | 32.4 (25.8-39.6) | 19.7 (14.3-26.1) | 35.6 (28.8-42.9) |
| Korangi | 64 | 26.6 (16.3-39.1) | 17.2 (8.9-28.7) | 26.6 (16.3-39.1) |
| Malir | 125 | 24.8 (17.5-33.3) | 10.4 (5.7-17.1) | 26.4 (18.9-35.0) |
| Kemari | 17 | 52.9 (27.8-77.0) | 35.3 (14.2-61.7) | 52.9 (27.8-77.0) |
| **Female gender (n=398)** | | | | |
| South | 19 | 31.6 (12.6-56.5) | 26.3 (9.2-51.2) | 31.6 (12.6-56.5) |
| West | 18 | 22.2 (6.4-47.6) | 22.2 (6.4-47.6) | 22.2 (6.4-47.6) |
| East | 104 | 31.7 (22.9-41.6) | 23.1 (15.4-32.4) | 31.7 (22.9-41.6) |
| Central | 88 | 33.0 (23.3-43.4) | 25.0 (16.4-35.4) | 35.2 (25.3-46.1) |
| Korangi | 47 | 48.9 (34.1-63.9) | 40.4 (26.4-55.7) | 51.1 (36.1-65.9) |
| Malir | 96 | 41.7 (31.7-52.2) | 32.3 (23.1-42.6) | 41.7 (31.7-52.2) |
| Kemari | 26 | 34.6 (17.2-55.7) | 23.1 (8.9-43.7) | 34.6 (17.2-55.7) |

| **Supplementary Table 4: Sensitivity, specificity, and level of agreement of NCEP ATP III and modified NCEP ATP III for MetS using IDF as standard definition stratified by age and gender** | | | | |
| --- | --- | --- | --- | --- |
| **Definition** | **IDF** | | | |
|  | **Sensitivity** | **Specificity** | **Kappa Index** | **p-value** |
|  | **% (95% CI)** | **% (95% CI)** |  |  |
| **Age <30 years (n=208)** | | | | |
| NCEP ATP III | 46.8 (32.1-61.9) | 96.3 (92.1-98.6) | 0.503 | <0.001 |
| Modified NCEP ATP III | 100 (92.5-100) | 95.6 (91.3-98.2) | 0.909 | <0.001 |
| **Age 30-50 years (n=607)** | | | | |
| NCEP ATP III | 66.2 (59.2-72.7) | 97.5 (95.5-98.8) | 0.687 | <0.001 |
| Modified NCEP ATP III | 100 (98.2-100) | 98.3 (96.5-99.3) | 0.974 | <0.001 |
| **Age >50 years (n=250)** | | | | |
| NCEP ATP III | 63.2 (52.6-72.8) | 94.8 (90.1-97.8) | 0.614 | <0.001 |
| Modified NCEP ATP III | 100 (96.2-100) | 96.8 (92.6-98.9) | 0.958 | <0.001 |
| **Male Gender (n=667)** | | | | |
| NCEP ATP III | 56.3 (49.1-63.3) | 96.6 (94.5-98.0) | 0.589 | <0.001 |
| Modified NCEP ATP III | 100 (98.2-100) | 96.6 (94.5-98.0) | 0.944 | <0.001 |
| **Female Gender (n=398)** | | | | |
| NCEP ATP III | 71.5 (63.4-78.7) | 96.8 (93.9-98.6) | 0.719 | <0.001 |
| Modified NCEP ATP III | 100 (97.5-100) | 98.8 (96.6-99.8) | 0.987 | <0.001 |
